# Single-Atom Anchored g-C_3_N_4_ Monolayer as Efficient Catalysts for Nitrogen Reduction Reaction

**DOI:** 10.3390/nano13081433

**Published:** 2023-04-21

**Authors:** Huadou Chai, Weiguang Chen, Zhen Feng, Yi Li, Mingyu Zhao, Jinlei Shi, Yanan Tang, Xianqi Dai

**Affiliations:** 1 School of Physics, Henan Normal University, Xinxiang 453007, China; chd916@163.com; 2 College of Physics and Electronic Engineering, Zhengzhou Normal University, Zhengzhou 450044, China; 3 School of Materials Science and Engineering, Henan Institute of Technology, Xinxiang 453000, China

**Keywords:** nitrogen reduction, single-atom catalytic, density functional theory, free energy, spin electrons distribution

## Abstract

Electrochemical N_2_ reduction reaction (NRR) is a promising approach for NH_3_ production under mild conditions. Herein, the catalytic performance of 3d transition metal (TM) atoms anchored on s-triazine-based g-C_3_N_4_ (TM@g-C_3_N_4_) in NRR is systematically investigated by density functional theory (DFT) calculations. Among these TM@g-C_3_N_4_ systems, the V@g-C_3_N_4_, Cr@g-C_3_N_4_, Mn@g-C_3_N_4_, Fe@g-C_3_N_4_, and Co@g-C_3_N_4_ monolayers have lower ΔG(*NNH) values, especially the V@g-C_3_N_4_ monolayer has the lowest limiting potential of −0.60 V and the corresponding limiting-potential steps are ${*}^{}N_{2}+H^{+}+e^{-}={*}^{}NNH$ for both alternating and distal mechanisms. For V@g-C_3_N_4_, the transferred charge and spin moment contributed by the anchored V atom activate N_2_ molecule. The metal conductivity of V@g-C_3_N_4_ provides an effective guarantee for charge transfer between adsorbates and V atom during N_2_ reduction reaction. After N_2_ adsorption, the p-d orbital hybridization of *N_2_ and V atoms can provide or receive electrons for the intermediate products, which makes the reduction process follow acceptance-donation mechanism. The results provide an important reference to design high efficiency single atom catalysts (SACs) for N_2_ reduction.

## 1. Introduction

Ammonia (NH_3_) is not only an essential substance to produce fertilizers, explosives, dyes, and pharmaceuticals, but also an important clean energy carrier [1,2,3,4]. The growing demand of NH_3_ has spurred researchers to seek more efficient artificial nitrogen (N_2_) fixation. Presently, large-scale production of NH_3_ mainly depends on the Haber–Bosch process. However, the process not only needs to operate under harsh conditions (300–500 °C and 200–300 atm) [5], but also requires huge energy input, and simultaneously generates a large number of greenhouse gases carbon dioxide (CO_2_) [6,7,8]. Considering energy consumption and environmental protection, researchers hope to find a method for converting N_2_ to NH_3_ using renewable energy, no polluting emissions, and mild operating conditions.

Electrochemical N_2_ reduction reaction (NRR), a promising approach for sustainable NH_3_ production under ambient conditions, has received extensive and increasing attention both in experiment and theoretic studies [9,10,11]. However, the strong bonding energy of N≡N triple bond (945 KJ/mol) and the weak adsorption of nonpolarized N_2_ are two major challenges in the NRR process [12,13,14]. Therefore, an efficient catalyst for N_2_ activation and reduction is urgent to improve NRR activity. On the other hand, the transition metal can accept the lone pair electrons of the N_2_ molecule and weak the N≡N triple bond [15,16,17]. Dispersing metal atoms on suitable supporting materials can not only provide more active sites, but also regulate the electronic properties of substrate and enhance the catalytic efficiency. Two-dimensional materials (2D) are widely used as catalyst substrates for N_2_ reduction due to their excellent chemical stability, thermal and electrical properties, and the ability to construct defect active sites through surface functionalization [18,19,20,21]. 

In recent years, transition metal single atom catalysts (SACs) constructed by anchoring single atom to two-dimensional materials have gained increasing attentions for the electrocatalysis applications and have shown excellent catalytic performances in NRR [22,23,24,25]. To avoid diffusion and agglomeration of metal atoms on the substrate, vacancies are usually constructed in the substrate to increase the stability of TM atom, or a two-dimensional material with a pore structure is used as the supporting substrate [26,27]. For example, the anchored Mo and Au on the N-doped porous carbon exhibited excellent NRR catalytic performance [28]. The Mo-embedded C_2_N monolayer has been demonstrated a very high NRR catalytic activity with the onset potential (U_onset_) of 0.17 V by Zhao et al. [29]. Thus, selecting the right metal atom and anchoring it to a suitable substrate with a pore structure can not only prevent the diffusion and agglomeration of the metal atom, but also higher catalytic activity can be obtained by the d-orbital electrons of metal atom inducing the π-back donation.

Graphitic carbon nitride (g-C_3_N_4_) has recently attracted great attentions because of its good physicochemical stability and superior properties [30,31]. tri-s-triazine-based g-C_3_N_4_ with large pore and s-triazine-based g-C_3_N_4_ with small pore are two common structures [32]. The uniform distribution of pore sites in both structures provide uniform nitrogen coordinators to capture metal atoms. Many studies [33,34] have shown that metal atoms anchored at tri-s-triazine-based g-C_3_N_4_ have excellent NRR performance. For example, Zhao et al. have found W-anchored tri-s-triazine-based g- C_3_N_4_ exhibits a high catalytic activity toward NRR with a limiting potential of −0.35 V [33]. Wang et al. have found that B/g-C_3_N_4_ can reduce N_2_ to NH_3_ with a lower onset potential (0.20 V) [34]. For s-triazine-based g-C_3_N_4_, it also has a pore structure surrounded by three nitrogen atoms and can be used as a metal-free substrate to anchor metal atoms. Hu et al. [35] have found that V@g-C_3_N_4_ with lying-on adsorbed N_2_ pattern has the lowest limiting potential of −0.79 V. However, implicit solvation model was not used to simulate the electrolyte solution, and the origin of the catalytic activity has not been explored in detail. It is necessary to systematically study the N_2_ reduction process in the electrolyte solution and explore the origin of the catalytic activity by anchoring single TM atom on s-triazine-based g-C_3_N_4_ (TM@g-C_3_N_4_).

Inspired by the above studies, the NRR catalytic behaviors of TM (TM = Sc, Ti, V, Cr, Mn, Fe, Co, Ni, Cu) atoms on intrinsic s-triazine-based g-C_3_N_4_ have been systematically investigated by density functional theory (DFT) theory. Theoretical calculations show that TM (V, Cr, Mn, Fe, Co) atoms anchored at g-C_3_N_4_ exhibits better NRR activities than Ru (0001) surface. V@g-C_3_N_4_ monolayer possesses the lowest limiting potential of −0.60 V, which is lower than that calculated by Hu et al. [35], and potential-limiting step (PLS) is *N_2_-*NNH for both alternating and distal mechanisms. The charge density difference, spin electrons distribution, density of states (DOS), the variation of charge transfer, D_V-N_, L_N-N_, and M_tot_ are used to explore the origin of the excellent catalytic performance. These results provide an important reference for the research of s-triazine-based g-C_3_N_4_ SACs.

## 2. Computational Methods

DFT methods are performed using the Vienna Ab Initio Simulation Package (5.4.4, 2017, VASP Software GmbH, Vienna, Austria) [36,37]. The exchange-correlation potentials are described through the Perdew–Burke– Ernerhof (PBE) parametrization within the generalized gradient approximation (GGA) [38,39]. The DFT-D3 method is utilized to describe the weak van der Waals (vdW)-like interaction [40]. The kinetic energy cutoff of plane-wave expansion is set to 520 eV and a vacuum space of 20 Å is inserted in the z-direction to eliminate the interaction between adjacent periodic units [41]. A 3 × 3 × 1 supercell is constructed. For geometrical optimizations, a 1 × 1 × 1 Monkhorst–Pack mesh of k-points is employed to sample the first Brillouin zero. A finer 5 × 5 × 1 k-points grid is chosen for the density of states (DOS). The convergence tolerances for the force and energy are set to 0.02 eV/Å and 10^−5^ eV, respectively. Bader charge is used analyze electron transfer [42].

The adsorption energies (E_ads_) of single TM atom and N_2_ molecule on substrate are calculated by the following formula:

(1) $$E_{ads}=E_{{adsorbate@substrate}_{}}-E_{substrate}-E_{adsorbate}$$

where $E_{{adsorbate@substrate}_{}}$, $E_{substrate}$, and $E_{adsorbate}$ are the total energies of adsorbate @substrate, substrate, and an isolate adsorbate, respectively. Negative E_ads_ indicates that the adsorbate can be stably bond to the substrate.

The cohesive energy (E_coh_) is calculated by the following formula:

(2) $$E_{coh}=E_{TM}-E_{TM,bulk}$$

where $E_{TM}$ and $E_{TM,bulk}$ are the energies of an isolated TM atom and bulk metal, respectively.

Computational hydrogen electrode (CHE) mode [43] is used to calculate the reaction free energy (ΔG). A solvation model is used to simulate the solution environment [44], and a relative permittivity of 80 is set for water [45]. The ΔG is evaluated as follows:

(3) $$\Delta G={\Delta E+\Delta ZPE}_{}-T\Delta S+\Delta G_{U}+\Delta G_{PH}$$

where ΔE is the reaction energy directly obtained by DFT calculations, ΔZPE and ΔS are the changes of zero-point energy (ZPE) and entropy computed from the vibrational frequencies at 298.15 K. $\Delta G_{U}$ is the free energy contribution with applied potential U, ΔG_U_ = eU, where e and U are the number of electrons transferred and the applied electrode potential. $\Delta G_{pH}=k_{B}\times pH\times ln10$, where pH = 0 in this work for simplicity. The limiting potential (U_L_) is defined as $U_{L}=-\Delta G_{max}/e$.

Using the CHE model, the lowest $∆G\left(pH,U\right)$ was evaluated. The ∆G(pH,U) values can be projected onto the (pH,U) plane of lowest Gibbs free energy, resulting in a 2D plot with pH and U descriptors to build the Pourbaix diagram [46]. The Pourbaix diagrams can provide the reliability electrocatalytic reaction results under certain conditions of pH and potential U.

## 3. Results and Discussion

### 3.1. Structural and Electronic Properties of TM@g-C_3_N_4_

**Figure 1:**
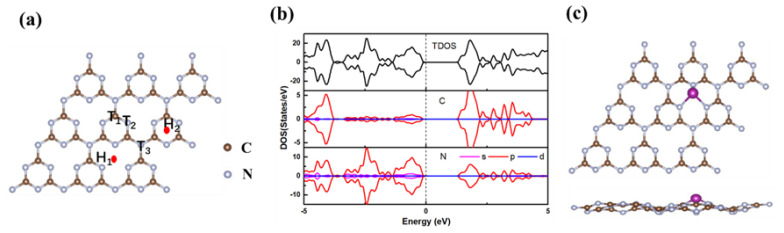
(**a**) Optimized structure and (**b**) DOS of s-tri-based g-C_3_N_4_ monolayer, (**c**) stable configuration of Mn@g-C_3_N_4_.

Optimized structure of s-triazine-based g-C_3_N_4_ monolayer is displayed in Figure 1a. It contains two C-N bonds, the bond lengths d_C-N_ are 1.33 Å and 1.46 Å, respectively. The optimized lattice constant is g-C_3_N_4_ is 4.78 Å, which is consistent with previous theoretical results [47,48]. The symmetrical distribution of spin-up and spin-down electrons in Figure 1b indicates that the g-C_3_N_4_ is a non-magnetic semiconductor. TM (Sc-Cu) atom is anchored at five high-symmetry sites (Figure 1a) to investigate the stable configurations, namely, the top sites of C (T_1_), pyridine N (T_2_), and graphite N (T_3_), hollow sites above the three pyridine N (H_1_) and the triazine ring (H_2_). Taking Mn atom as an example, the calculated adsorption energies (E_ads_) are −5.16 eV, −6.01 eV, −4.49 eV, −6.20 eV, −3.70 eV, respectively, and the H_1_ site is the most stable adsorption site with the lowest E_ads_ −6.20 eV. All the TM atoms have a same stable adsorption site H_1_. The stable configuration of Mn atom adsorbed at H_1_ site is shown in Figure 1c. The adsorption of Mn atom causes the distortion of g-C_3_N_4_ monolayer, but no bond breaks. The other configurations of TM@g-C_3_N_4_ are displayed in Figure S1.

The calculated adsorption energies (E_ads_), energy difference between adsorption energy and cohesive energy (ΔE), total magnetic moments (M_tot_), the charge (ΔQ) transferred from TM atoms to g-C_3_N_4_ monolayer, and the electronic structures (ES) of TM@g-C_3_N_4_ are shown in Table 1. The premise of good catalytic performance of SAC is that single metal atom has suitable E_ads_ on the substrate. The calculated E_ads_ values of TM@g-C_3_N_4_ are in the range of −9.55~−4.76 eV, which indicates that TM atoms can bind stably to the substrate. At the same time, the energy difference (ΔE) between the adsorption energy (E_ads_) and the cohesive energy (E_coh_) is calculated to investigate the aggregation of TM atoms on g-C_3_N_4_. The value of ΔE is in the range of −5.36~−0.92 eV, and a negative value of ΔE means that the adsorption of metal atoms on g-C_3_N_4_ is stronger than the cohesive of atoms. Hence, the aggregation of TM atoms on g-C_3_N_4_ can be suppressed efficiently. TM (Sc-Cu) atoms anchored to g-C_3_N_4_ holes have good stability.

The spin densities distribution of TM@g-C_3_N_4_ are shown in Figure S2. It can be seen that spin electrons are located at TM (TM= Sc, Ti, V, Cr, Mn, Fe, Co, Cu) and surrounding atoms. Compared with Figure 1b, it is obvious that the anchored TM atoms (TM= Sc, Ti, V, Cr, Mn, Fe, Co, Cu) induce the magnetic moment of TM@g-C_3_N_4_. V@g-C_3_N_4_ has the most spin densities, which is consistent with the largest moment (4.88 μ_B_) in Table 1.

As is well known, the anchored TM atoms can regulate the electronic structure of substrate, and the catalytic performance of the catalyst is closely related to the electronic structure. Hence, the total density of states (TDOS) of TM@g-C_3_N_4_ and partial DOS (PDOS) are calculated and shown in Figure S3. It is seen that the anchored TM (TM=Sc, Ti, V, Cr, Mn, Fe, Co, Cu) atoms induce the asymmetry of spin-up and spin-down electrons of TM@g-C_3_N_4_, which is consistent with the spin electrons distribution in Figure S2. Second, the adsorption of Cr, Mn, Co, and Ni atoms retain the semiconductor property, but the band gap decreases from 1.57 eV to 0.38 eV, 0.46 eV, 0.58 eV, and 1.23 eV, respectively. The TDOSs of Ti@g-C_3_N_4_, V@g-C_3_N_4_, and Fe@g-C_3_N_4_ exhibit metallic properties and many electronic states are located near the Fermi level, which ensures a rapid transfer of charge in the reaction.

### 3.2. Adsorption of N_2_ Molecule

It is well known that the N_2_ adsorption on the catalytic is the first step to investigate the NRR performance, which reflects the sensitivity of the catalyst and determines the most favorable adsorption manner. Stable adsorption of N_2_ is a prerequisite for the subsequent reaction to generate NH_3_.

**Figure 2:**
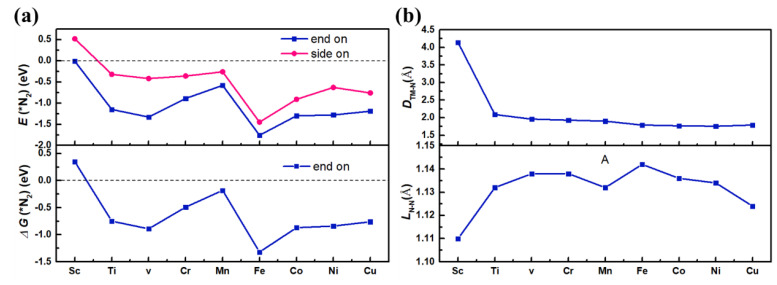
(**a**) E_ads_(*N_2_) and ΔG(*N_2_) of an N_2_ molecule on TM@g-C_3_N_4_, (**b**) D_TM-N_ and L_N-N_ of TM@g-C_3_N_4_.

The adsorption energies [E_ads_(*N_2_)] of TM@g-C_3_N_4_ (TM = Sc-Cu) are illustrated in Figure 2a. For TM@g-C_3_N_4_ (TM = Ti, V, Cr, Mn, Fe, Co, Ni, Cu), both end-on and side-on configurations can exist, while E_ads_(*N_2_) (−1.76~−0.58 eV) of end-on adsorption N_2_ molecule are lower than those (−1.45~−0.26 eV) of side on patterns, which means that end-on patterns are more energetically favorable than the side-on patterns. Therefore, in the following studies of this paper, only the favorable end-on adsorption manner is discussed. These results indicate that the TM atoms (TM = Ti, V, Cr, Mn, Fe, Co, Ni, Cu) anchored at g-C_3_N_4_ are active sites and have strong ability to capture N_2_ molecules. This result is consistent with the result calculated by Hu et al. [35] except for Ti atom.

In order to further study the adsorption of N_2_ on TM@g-C_3_N_4_, the distance between TM atom and the N_2_ molecule (D_TM-N_), and N-N bond length (L_N-N_) are displayed in Figure 2b. The D_TM-N_ (TM = Ti, V, Cr, Mn, Fe, Co, Ni, Cu) are in the range of 1.76~2.09 Å and the L_N-N_ are stretched to 1.13~1.14 Å, which indicates that the N≡N triple bonds are weakened and good for reduction reaction. For Sc@g-C_3_N_4_, the adsorption energies for end-on and side-on patterns are −0.01 eV and 0.52 eV, the D_Sc-N_ is 4.14 Å and the L_N-N_ is 1.11 Å, which means that Sc@g-C_3_N_4_ has poor NRR activity. TM@g-C_3_N_4_ (TM = Ti, V, Cr, Mn, Fe, Co, Ni, Cu) will be considered as SAC candidates in the following studies.

### 3.3. N_2_ Electrocatalytic Reduction Reaction

The second criterion for good NRR catalyst is that it has a lower limiting potential in the N_2_ reduction reaction. To evaluate the catalytic activities of TM@g-C_3_N_4_ (TM = Ti, V, Cr, Mn, Fe, Co, Ni, Cu), the NRR mechanisms are investigated to obtain the potential-limiting step (PLS) and limiting potential.

Theoretical and experimental studies [49] on NRR show that the end-on adsorbed N_2_ molecule on TM@g-C_3_N_4_ (TM = Ti, V, Cr, Mn, Fe, Co, Ni, Cu) can be reduced to NH_3_ via a distal or alternating mechanism. In the distal mechanism, the proton (H^+^) continuously attacks the terminal N atom until the first NH_3_ molecule is released and then the other. While for the alternating mechanism, the proton (H^+^) alternating bonds the two N atoms to release NH_3_ molecule. 

**Figure 3:**
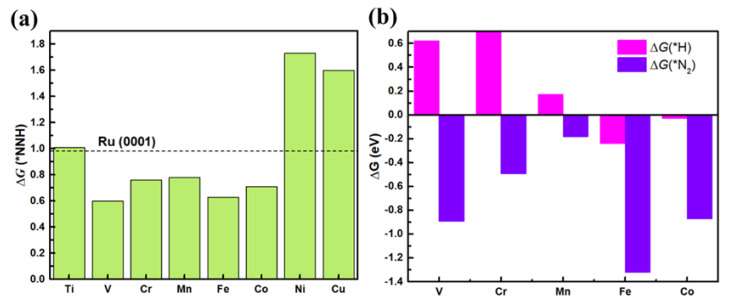
(**a**) The reaction free energy ΔG(*NNH), (**b**) absorption free energies of N_2_ molecules ΔG(*N_2_) and H atoms ΔG(*H) on the TM@g-C_3_N_4_ (TM = V, Cr, Mn, Fe, Co).

Previous studies suggest that the first H^+^ reaction ${*}^{}N_{2}+H^{+}+e^{-}={*}^{}NNH$ is usually the potential-limiting step (PLS) for NRR [16,50]. The free energy barrier ΔG(*NNH) is calculated and displayed in Figure 3a. It can be seen that the ΔG(*NNH) values of V@g-C_3_N_4_ (0.60 eV), Cr@g-C_3_N_4_ (0.76 eV), Mn@g-C_3_N_4_ (0.78 eV), Fe@g-C_3_N_4_ (0.63 eV), and Co@g-C_3_N_4_ (0.71 eV) are lower than that on Ru (0001) surface. On the other hand, since the hydrogen evolution reaction (HER) is a major competitor for NRR [17,51], the calculated free energies change of H [ΔG(*H)] are shown in Figure 3b. It is seen that ΔG(*H) are positive for V@g-C_3_N_4_, Cr@g-C_3_N_4_, and Mn@g-C_3_N_4_, which means that H atom cannot be adsorbed on V, Cr and Mn atoms. For Fe@g-C_3_N_4_ and Co@g-C_3_N_4_, the ΔG(*H) is negative, while the values of ΔG(*N_2_) are lower than ΔG(*H) and they prefer to adsorb N_2_ molecule. In conclusion, V, Cr, Mn, Fe, and Co atoms anchored by g-C_3_N_4_ are selected as potential SACs for NRR by the second criterion.

In order to explore the whole N_2_ reduction process, it is necessary to explore the PLS of the process. The PLS dominates the catalytic efficiency of the catalyst for NRR. The results show that V@g-C_3_N_4_ is the best catalyst with the lowest ΔG(*NNH) value 0.60 eV compared with Cr@g-C_3_N_4_, Mn@g-C_3_N_4_, Fe@g-C_3_N_4_, and Co@g-C_3_N_4_. The whole free energy diagrams of V@g-C_3_N_4_ during NRR process is further investigated with the favorable end-on adsorption manner.

**Figure 4:**
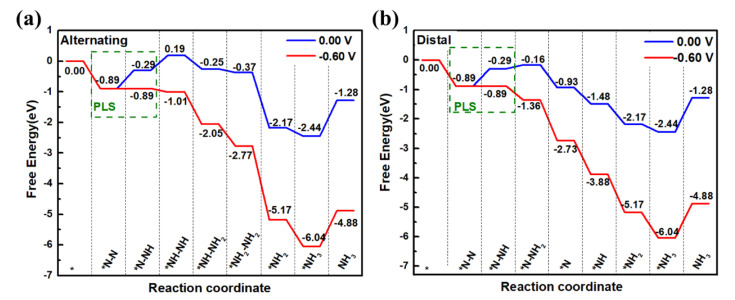
NRR free energy diagrams via (**a**) alternating (**b**) distal mechanisms for V@g-C_3_N_4_.

The NRR free energy diagrams of V@g-C_3_N_4_ are displayed in Figure 4. Optimized adsorption structures of intermediate via the alternating and distal pathways are depicted in Figure S4. The free energy diagrams via alternating and distal mechanisms are very similar. During N_2_ reduction process, the N_2_ adsorption and protonation into *NNH along the two mechanisms are same. The adsorption process of $*+N_{2}={*}^{}N_{2}$ is downhill with ΔG value −0.89 eV, which shows that V@g-C_3_N_4_ can spontaneously adsorb the N_2_ molecule. The step of ${*}^{}N_{2}+H^{+}+e^{-}={*}^{}NNH$ is uphill with ΔG value 0.60 eV, which means that the protonation process is endothermic. The second proton-electron may bond with another N atom of *N_2_ molecule to generate *NHNH (alternating mechanism) or the same N atom (distal mechanism) to generate *NNH_2_, and the hydrogenation in both ways are both uphill with ΔG value 0.48 eV and 0.13 eV, respectively. In the following steps of two mechanisms, ΔG values are all negative. For alternating mechanism in Figure 4a, the calculated ΔG values are −0.89, 0.60, 0.48, −0.44, −0.12, −1.81 eV, and −0.27 eV, respectively. The first protonation step has the highest ΔG values and is confirmed to be the PLS with an uphill value 0.60 eV. When the U_L_ is applied to −0.60 V, all the reaction steps are exothermic, therefore, the limiting potential is −0.60 V. For distal mechanism in Figure 4b, the ΔG values are −0.89, 0.60, 0.13, −0.77, −0.55, −0.70, and −0.27 eV, respectively, and it is obvious that it has the same PLS and U_L_ as the alternating mechanism. The calculated limiting potentials of V@g-C_3_N_4_ are lower than those calculated by Hu et al., where the limiting potential of V@g-C_3_N_4_ with lying-on adsorbed N_2_ is −0.79 V, while that of standing-on pattern is −0.93 V [35]. The final desorption of the second *NH_3_ molecule requires the absorption energy of 1.16 eV, and it can also be further protonated to form NH_4_^+^ [52].

Pourbaix diagrams can provide an effective guidance for the electrocatalytic reactions, which will not be discussed in this article.

### 3.4. Origin of Catalytic Activity

**Figure 5:**
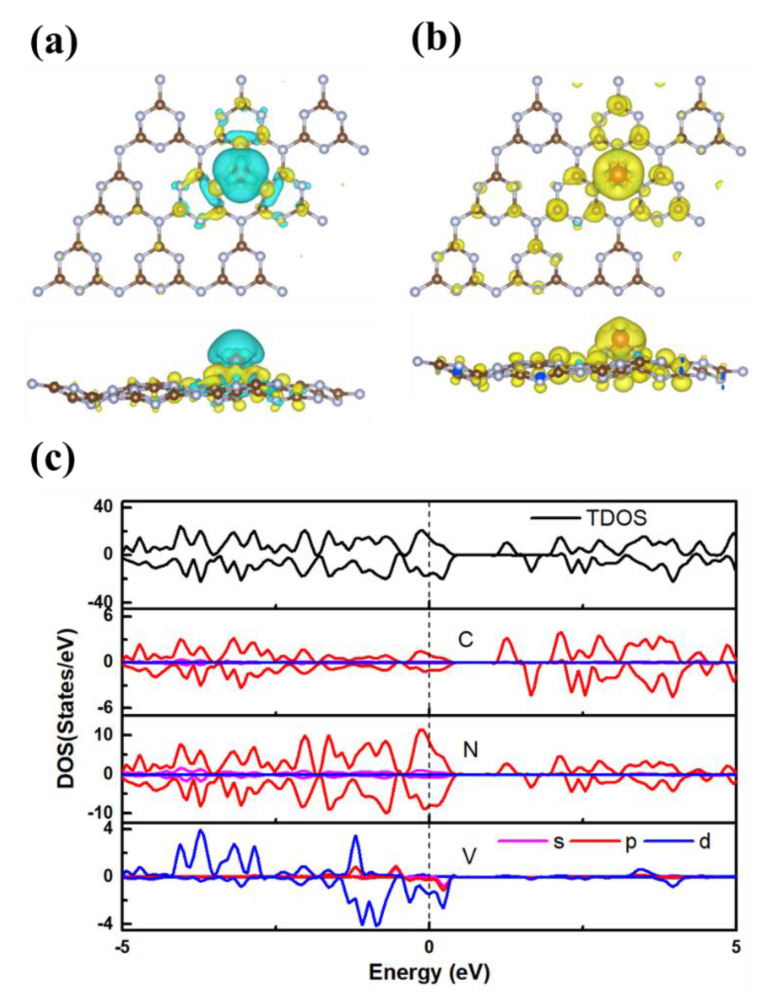
(**a**) Charge density difference with isosurface 0.004 e/Bohr^3^, the yellow and blue areas represent the accumulation and depletion of electrons, (**b**) spin density, the yellow and blue regions represent the spin-up and spin-down states, (**c**) DOS of V@g-C_3_N_4_, the Fermi level is set to 0 eV.

To explore the excellent catalytic efficiency of V@g-C_3_N_4_, the electronic properties, charge density difference, and spin electrons distribution are investigated. The charge density difference is shown in Figure 5a. It can be seen that the electron densities of the three N atoms anchoring the V atom increases, and Bader charge analysis finds that the V atom loses 1.92 e, which makes the V atom be a good active site for trapping N_2_ molecule. Figure 5b shows that the spin density is mainly located at the V atom, which makes V@g-C_3_N_4_ possess a total spin moment of 4.88 μ_B_. These results prove the conclusion that the magnetism of the catalyst can increase its activity. The density of states (DOS) is presented in Figure 5c. Different from the intrinsic *s*-triazine-based g-C_3_N_4_ monolayer of direct bandgap semiconductor in Figure 1b [47,48], V@g-C_3_N_4_ exhibits a metallic property owing to the V atom. The spin moment is vital to activate N_2_ molecule, and the excellent electrical conductivity is essential to ensure good charge transfer for efficient electroreduction reaction [48,53]. Both of them play an important role in the outstanding NRR catalytic performance of V@g-C_3_N_4_. 

**Figure 6:**
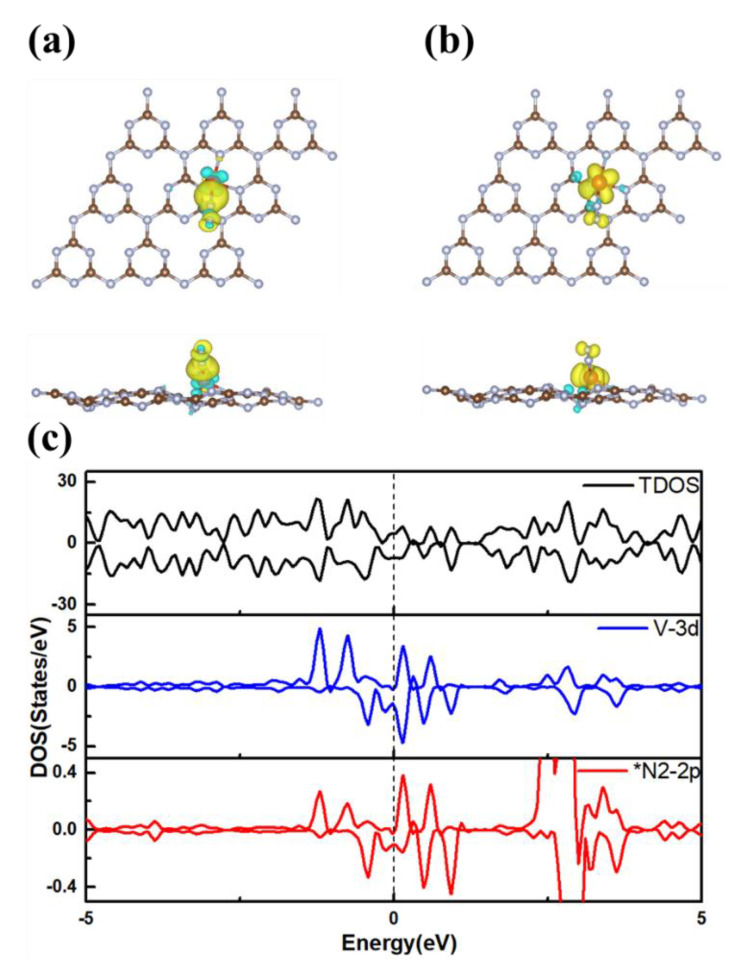
(**a**) Charge density difference with isosurface 0.004 e/Bohr^3^, the yellow and blue areas represent the accumulation and depletion of electrons, (**b**) spin density, the yellow and blue regions represent the spin-up and spin-down states, (**c**) DOS of N_2_/V@g-C_3_N_4_, the Fermi level is set to 0 eV.

In order to further explore the understanding NRR catalytic performance of V@g-C_3_N_4_, the charge density difference and spin electrons distribution after N_2_ adsorption are calculated. As shown in Figure 6a, the electrons accumulate near the *N_2_ molecule, and it is confirmed by Bader charge analysis that 0.42 e transferred from V atom to the N_2_ molecule. The spin electrons distribution in Figure 6b reveals that after N_2_ adsorption, some spin moment on V atom can be transferred to the N_2_ molecule, so that the N_2_/V@g-C_3_N_4_ only possesses a magnetic moment 1.00 μ_B_. The change in charge and spin magnetic moment drives its further activation and reactions. The DOS of N_2_/V@g-C_3_N_4_ are displayed in Figure 6c. Obvious overlap between the *N_2_-2p and V-3d orbitals around the Fermi energy happens, in which the empty d orbitals of V can accept the lone-pair electrons in N_2_, at the same time, the occupied d orbitals can donate electrons to the antibonding orbitals of N_2_, and this process follows acceptance-donation mechanism. 

**Figure 7:**
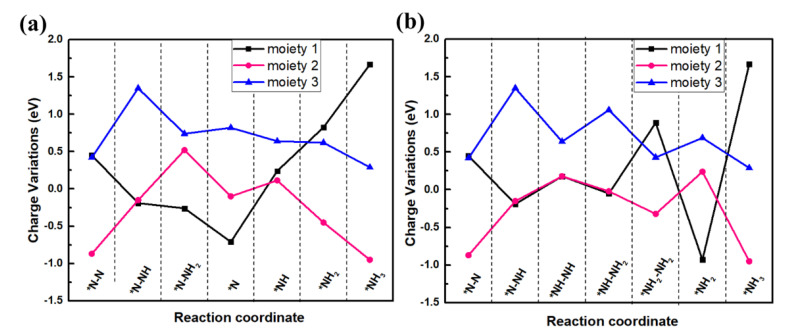
Charge variation diagrams of adsorbates via (**a**) distal and (**b**) alternating mechanism.

To further explore the charge transferred among intermediate adsorbent, the variations of the charge transferred between them are studied. Based on the previous studies [54,55], N_x_H_y_/V@g-C_3_N_4_ is divided into three parts (Figure S5): g-C_3_N_4_ substrate without V and the three N atoms bonded with it (moiety 1), V-3N (moiety 2), and the adsorbed intermediate N_x_H_y_ (moiety 3). The charge variations along distal and alternating mechanisms are shown in Figure 7 (the charge variation is the charge difference of each moiety between the present step and the previous step). For N_2_ adsorption on V@g-C_3_N_4_ monolayer in Figure 7a, V-3N offers 0.42 e and 0.45 e to *N_2_ and g-C_3_N_4_, respectively. During the protonation process ${*}^{}N_{2}+H^{+}+e^{-}={*}^{}NNH$, V-3N and g-C_3_N_4_ substrate provide 0.15 e and 0.19 e, respectively. However, during the second protonation process ${*}^{}NNH+H^{+}+e^{-}={*}^{}{NNH}_{2}$, V-3N gains 0.52 e from the substrate and *NNH_2_. In the following hydrogenation and reduction steps, obvious charge fluctuation occurs for the three moieties, which means that V-3N is the charge transferred medium between N_x_H_y_ and g-C_3_N_4_ substrate, providing or receiving electrons to the adsorbed intermediate. The above results once again prove that the excellent catalytic performance of V@g-C_3_N_4_ in NRR process follows the acceptance-donation mechanism. The charge variation in the alternating mechanism shown in Figure 7b is similar to those the distal mechanism.

**Figure 8:**
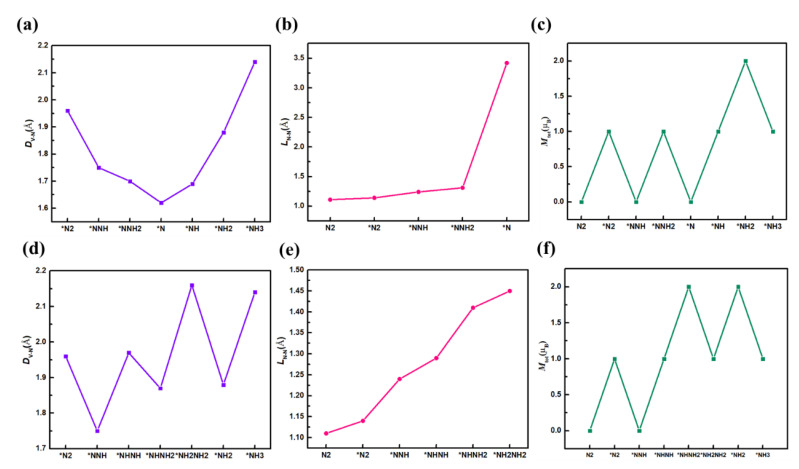
*D*_V-N_, *L*_N-N_ and total magnetic moment (*M*_tot_) of adsorbates on V@g-C_3_N_4_ via an (**a**–**c**) distal mechanism and (**d**–**f**) alternating mechanism.

In order to explore the change of adsorbates configuration and magnetic properties during the reduction of N_2_, the V-N distance (D_V-N_), N-N bond length (L_N-N_), and the total magnetic moments (M_tot_) are examined and displayed in Figure 8. In Figure 8a, D_V-N_ along distal pathway decreases during protonation and reaches a minimum when the first NH_3_ molecule is released, and then continuously increases. While in alternating mechanism, when the proton (H^+^) attacks the distal N atom, D_V-N_ decreases, and when it attacks the N atom bonded to V, D_V-N_ increases. D_V-N_ increases in a zigzagging fluctuation way during the whole reduction process. For L_N-N_, as shown in Figure 8b, it is stretched continuously until the first NH_3_ is released for both distal and alternating mechanisms. Figure 8c shows the magnetic moments of the N_x_H_y_, and it can be seen that the protonation process makes the M_tot_ show zigzag fluctuations for both mechanisms. These results provide strong evidence for the excellent catalytic performance of V@g-C_3_N_4_.

## 4. Conclusions

In this paper, the catalytic performance of 3d TM atoms anchored on s-triazine-based g-C_3_N_4_ (TM@g-C_3_N_4_) for N_2_ reduction is systematically investigated. The results show that N_2_ molecules can be stably adsorbed on TM@g-C_3_N_4_ (TM=Ti, V, Cr, Mn, Fe, Co, Ni, Cu), and the end-on modes are more energetically favorable than the side-on modes. The ΔG(*NNH) values indicate that TM@g-C_3_N_4_ (TM = V, Cr, Mn, Fe, Co) are potential NRR candidates with lower ΔG(*NNH) value than that on Ru (0001) surface. The free energy diagrams show that V@g-C_3_N_4_ exhibits the highly catalytic performance for NRR with a limiting potential −0.60 V, and the PLS is the first protonation of *N_2_ for both alternating and distal mechanisms. In order to explore the origin of excellent NRR performance of V@g-C_3_N_4_, the charge density difference, spin electrons distribution and DOS plots before and after N_2_ adsorption, and their corresponding charge variation of adsorbates during N_2_ reduction reaction are discussed. First, for V@g-C_3_N_4_, V atom as an active site provide 1.92 e and has the ability to trap N_2_ molecule. A spin moment of 4.88 μ_B_ of V@g-C_3_N_4_ contributed by V atom can activate N_2_ molecule. Second, after N_2_ adsorption, the strong interaction between *N_2_-2p and V-3d orbitals ensures the charge transferred during N_2_ reduction reaction. Third, the charge variation of moieties indicate that V-3N acts as a medium between adsorbates N_x_H_y_ and g-C_3_N_4_ substrate, which ensures the efficient reduction of N_2_. The D_V-N_, L_N-N_ and M_tot_ of N_x_H_y_/V@g-C_3_N_4_ are calculated to illustrate the change of adsorbate configurations and magnetic properties during the N_2_ reduction reactions, which provides an important evidence for the excellent catalytic performance of SACs TM@g-C_3_N_4_.

## Figures and Tables

**Table 1:** Adsorption energies (E_ads_, eV), energy difference (ΔE, eV) between adsorption energy and cohesive energy, total magnetic moments (M_tot_, μ_B_), the charge (ΔQ, e) transferred from the TM atoms to g-C_3_N_4_ monolayer, and the electronic structures (ES) (SM: semi-metallic, M: metallic and SC: semiconductor).

	E_ads_	ΔE	M_tot_	ΔQ	ES
Sc@g-C_3_N_4_	−9.55	−5.36	1.00	2.35	SM
Ti@g-C_3_N_4_	−8.26	−2.80	2.00	2.21	M
V@g-C_3_N_4_	−7.50	−2.13	4.88	1.92	M
Cr@g-C_3_N_4_	−5.97	−1.96	4.00	1.59	SC
Mn@g-C_3_N_4_	−6.20	−2.34	1.00	1.48	SC
Fe@g-C_3_N_4_	−5.70	−0.92	−0.26	1.35	M
Co@g-C_3_N_4_	−7.25	−1.89	1.00	1.02	SC
Ni@g-C_3_N_4_	−7.32	−2.42	0	0.94	SC
Cu@g-C_3_N_4_	−4.96	−1.45	1.00	0.86	SM

## Data Availability

The data presented in this study are available on a reasonable request from the corresponding author.

## Supplementary Materials

The following supporting information can be downloaded at: https://www.mdpi.com/article/10.3390/nano13081433/s1, Figure S1: Stable configurations of TM@ g-C3N4. Figure S2: Spin electrons distribution of TM@ g-C3N4. Figure S3: DOS of TM@ g-C3N4. Figure S4 Optimized adsorption structures of intermediate via the alternating and distal mechanisms. Figure S5. Diagram of the moiety 1(g-C3N4), moiety 2 (V-3N) and moiety 3 (the adsorbed intermediate NxHy).

## References

[1] Erisman J.W., Sutton M.A., Galloway J., Klimont Z., Winiwarter W. (2008). How a century of ammonia synthesis changed the world. Nat. Geosci..

[2] Schlögl R. (2003). Catalytic synthesis of ammonia—A “never-ending story”?. Angew. Chem. Int. Ed..

[3] Galloway J.N., Townsend A.R., Erisman J.W., Bekunda M., Cai Z., Freney J.R., Martinelli L.A., Seitzinger S.P., Sutton M.A. (2008). Transformation of the nitrogen cycle: Recent trends, questions, and potential solutions. Science.

[4] Canfield D.E., Glazer A.N., Falkowski P.G. (2010). The evolution and future of Earth’s nitrogen cycle. Science..

[5] Liu H. (2014). Ammonia synthesis catalyst 100 years: Practice, enlightenment and challenge. Chin. J. Catal..

[6] Kitano M., Kanbara S., Inoue Y., Kuganathan N., Sushko P.V., Yokoyama T., Hara M., Hosono H. (2015). Electride support boosts nitrogen dissociation over ruthenium catalyst and shifts the bottleneck in ammonia synthesis. Nat. Commun..

[7] Chen P. (2018). Across the board: Ping chen. ChemSusChem.

[8] McEnaney J.M., Singh A.R., Schwalbe J.A., Kibsgaard J., Lin J.C., Cargnello M., Jaramillo T.F., Nørskov J.K. (2017). Ammonia synthesis from N_2_ and H_2_O using a lithium cycling electrification strategy at atmospheric pressure. Energy Environ. Sci..

[9] Hao Y.C., Guo Y., Chen L., Shu M., Wang X.Y., Bu T., Gao W., Zhang N., Su X., Feng X. (2019). Promoting nitrogen electroreduction to ammonia with bismuth nanocrystals and potassium cations in water. Nat. Catal..

[10] Guo X., Gu J., Hu X., Zhang S., Chen Z., Huang S. (2020). Coordination tailoring towards efficient single-atom catalysts for N_2_ fixation: A case study of iron-nitrogen-carbon (Fe@N-C) systems. Catal. Today.

[11] Xiao B.B., Yang L., Yu L.B., Song E.H., Jiang Q. (2020). The VN_3_ embedded graphane with the improved selectivity for nitrogen fixation. Appl. Surf. Sci..

[12] Yang C., Zhu Y., Liu J., Qin Y., Wang H., Liu H., Chen Y., Zhang Z., Hu W. (2020). Defect engineering for electrochemical nitrogen reduction reaction to ammonia. Nano Energy..

[13] Chen C., Liang C., Xu J., Wei J., Li X., Zheng Y., Li J., Tang H., Li J. (2020). Size-dependent electrochemical nitrogen reduction catalyzed by monodisperse Au nanoparticles. Electrochim. Acta..

[14] Wu T., Zhu X., Xing Z., Mou S., Li C., Qiao Y., Liu Q., Luo Y., Shi X., Zhang Y. (2019). Greatly improving electrochemical N_2_ reduction over TiO_2_ nanoparticles by iron doping. Angew. Chem. Int. Ed..

[15] Honkala K., Hellman A., Remediakis I.N., Logadottir A., Carlsson A., Dahl S., Christensen C.H., Nørskov J.K. (2005). Ammonia synthesis from first-principles calculations. Science.

[16] Skulason E., Bligaard T., Gudmundsdóttir S., Studt F., Rossmeisl J., Abild-Pedersen F., Vegge T., Jo H., Nørskov J.K. (2012). A theoretical evaluation of possible transition metal electro-catalysts for N_2_ reduction. Phys. Chem. Chem. Phys..

[17] Wan Y., Xu J., Lv R. (2019). Heterogeneous electrocatalysts design for nitrogen reduction reaction under ambient conditions. Mater. Today.

[18] Jiao D., Liu Y., Cai Q., Zhao J. (2021). Coordination tunes the activity and selectivity of the nitrogen reduction reaction on single-atom iron catalysts: A computational study. J. Mater. Chem. A.

[19] Li H., Zhao Z., Cai Q., Yin L., Zhao J. (2020). Nitrogen electroreduction performance of transition metal dimers embedded into N-doped graphene: A theoretical prediction. J. Mater. Chem. A.

[20] Yang W., Huang H., Ding X., Ding Z., Wu C., Gates I., Gao Z. (2020). Theoretical study on double-atom catalysts supported with graphene for electroreduction of nitrogen into ammonia. Electrochim. Acta..

[21] Wu D., Lv P., Wu J., He B., Li X., Chu K., Jia Y., Ma D. (2023). Catalytic active centers beyond transition metals: Atomically dispersed alkaline-earth metals for the electroreduction of nitrate to ammonia. J. Mater. Chem. A.

[22] Liu K., Fu J., Zhu L., Zhang X., Li H., Liu H., Hu J., Liu M. (2020). Single-atom transition metals supported on black phosphorene for electrochemical nitrogen reduction. Nanoscale.

[23] Han M., Wang G., Zhang H., Zhao H. (2019). Theoretical study of single transition metal atom modified MoP as a nitrogen reduction electrocatalyst. Phys. Chem. Chem. Phys..

[24] Ma D., Zeng Z., Liu L., Huang X., Jia Y. (2019). Computational evaluation of electrocatalytic nitrogen reduction on TM single-, double-, and triple-atom catalysts (TM = Mn, Fe, Co, Ni) based on graphdiyne monolayers. J. Phys. Chem. C.

[25] Ou P., Zhou X., Meng F., Chen C., Chen Y., Song J. (2019). Single molybdenum center supported on N-doped black phosphorus as an efficient electrocatalyst for nitrogen fixation. Nanoscale..

[26] Zhang B., Fan T., Xie N., Nie G., Zhang H. (2019). Versatile applications of metal single-atom @ 2D material nanoplatforms. Adv. Sci..

[27] Zhu Y., Peng W., Li Y., Zhang G., Zhang F., Fan X. (2019). Modulating the electronic structure of single-atom catalysts on 2D nanomaterials for enhanced electrocatalytic performance. Small Methods.

[28] Han L., Liu X., Chen J., Lin R.., Liu H., Xin H. (2019). Atomically dispersed molybdenum catalysts for efficient ambient nitrogen fixation. Angew. Chem..

[29] Wang Z., Yu Z., Zhao J. (2018). Computational screening of a single transition metal atom supported on the C_2_N monolayer for electrochemical ammonia synthesis. Phys. Chem. Chem. Phys..

[30] Zhao Z., Sun Y., Dong F. (2015). Graphitic carbon nitride based nanocomposites: A review. Nanoscale.

[31] Liu J., Cheng B., Yu J. (2016). A new understanding of the photocatalytic mechanism of the direct Z-scheme g-C_3_N_4_/TiO_2_ heterostructure. Phys. Chem. Chem. Phys..

[32] Zhu B., Cheng B., Zhang L., Yu J. (2019). Review on DFT calculation of s-triazine-based carbon nitride. Carbon Energy.

[33] Chen Z., Zhao J., Cabrera C., Chen Z. (2019). Computational screening of efficient single-atom catalysts based on graphitic carbon nitride (g-C_3_N_4_) for nitrogen electroreduction. Small Methods.

[34] Ling C., Niu X., Li Q., Du A., Wang J. (2018). Metal-free single atom catalyst for N_2_ fixation driven by visible light. J. Am. Chem. Soc..

[35] Zhang L., Zhao W., Zhang W., Chen J., Hu Z. (2019). gt−C_3_N_4_ coordinated single atom as an efficient electrocatalyst for nitrogen reduction reaction. Nano. Res..

[36] Kresse G., Furthmüller J. (1996). Efficiency of ab-initio total energy calculations for metals and semiconductors using a plane-wave basis set. Comput. Mater. Sci..

[37] Kresse G., Furthmüller J. (1996). Efficient iterative schemes for ab initio total-energy calculations using a plane-wave basis set. Phys. Rev. B.

[38] Kresse G., Joubert D. (1999). From ultrasoft pseudopotentials to the projector augmented-wave method. Phys Rev. B.

[39] Perdew J., Burke K., Ernzerhof M. (1996). Generalized gradient approximation made simple. Phys. Rev. Lett..

[40] Grimme S. (2006). Semiempirical GGA-type density functional constructed with a long-range dispersion correction. J. Comput. Chem..

[41] Bermudez A., Jelezko F., Plenio M., Retzker A. (2011). Electron-mediated nuclear-spin interactions between distant nitrogen-vacancy centers. Phys. Rev. Lett..

[42] Chai H., Chen W., Li Y., Tang Y., Dai X. (2022). The adsorption properties and stable configurations of hydroxyl groups at Mo edge of MoS_2_ (100) surface. Mater. Chem. Phys..

[43] Nørskov J.K., Rossmeisl J., Logadottir A., Lindqvist L., Kitchin J.R., Bligaard T., Jónsson H. (2004). Origin of the overpotential for oxygen reduction at a fuel-cell cathode. J. Phys. Chem. B.

[44] Mathew K., Sundararaman R., Letchworth-Weaver K., Arias T.A., Hennig R.G. (2014). Implicit solvation model for density-functional study of nanocrystal surfaces and reaction pathways. J. Chem. Phys..

[45] Reda M., Hansen H.A., Vegge T. (2018). DFT study of stabilization effects on N-doped graphene for ORR catalysis. Catal. Today.

[46] López M., Exner K.S., Viñes F., Illas F. (2022). Computational Pourbaix diagrams for MXenes: A key ingredient toward proper theoretical electrocatalytic studies. Adv. Theory. Simul..

[47] Zhu B., Wageh S., Al-Ghamdi A., Yang S., Tian Z., Yu J. (2019). Adsorption of CO_2_, O_2_, NO and CO on s-triazine-based g-C_3_N_4_ surface. Catal. Today.

[48] Praus P. (2022). A brief review of s-triazine graphitic carbon nitride. Carbon Lett..

[49] Li X., Li Q., Cheng J., Liu L., Yan Q., Wu Y., Zhang X.H., Wang Z.Y., Qiu Q., Luo Y. (2016). Conversion of dinitrogen to ammonia by FeN_3_-embedded graphene. J. Am. Chem. Soc..

[50] Liu C., Li Q., Zhang J., Jin Y., MacFarlane D.R., Sun C. (2019). Conversion of dinitrogen to ammonia on Ru atoms supported on boron sheets: A DFT study. J. Mater. Chem. A.

[51] Suryanto B.H.R., Du H., Wang D., Chen J., Simonov A.N., MacFarlane D.R. (2019). Challenges and prospects in the catalysis of electroreduction of nitrogen to ammonia. Nat. Catal..

[52] Gao L., Wang F., Yu M.-a., Wei F., Qi J., Lin S., Xie D. (2019). A novel phosphotungstic acid-supported single metal atom catalyst with high activity and selectivity for the synthesis of NH_3_ from electrochemical N_2_ reduction: A DFT prediction. J. Mater. Chem. A.

[53] Zhao J., Chen Z. (2017). Single Mo atom supported on defective boron nitride monolayer as an efficient electrocatalyst for nitrogen fixation: A computational study. J. Am. Chem. Soc..

[54] Xu Z., Song R., Wang M., Zang X., Liu G., Qiao G. (2020). Single atom-doped arsenene as electrocatalyst for reducing nitrogen to ammonia: A DFT study. Phys. Chem. Chem. Phys..

[55] Xue Z., Zhang X., Qin J., Liu R. (2021). Anchoring Mo on C_9_N_4_ monolayers as an efficient single atom catalyst for nitrogen fixation. J. Energy Chem..

