# Supplementary material for: Single-Atom Anchored g-C3N4 Monolayer as Efficient Catalysts for Nitrogen Reduction Reaction
Source: Nanomaterials (Basel). 2023 Apr 21;13(8):1433. doi: 10.3390/nano13081433 (PMC10142710; doi:10.3390/nano13081433)
Supplement: Supplementary file 1 [file nanomaterials-13-01433-s001.zip › nanomaterials-2268367-supplementary.pdf]

nanomaterials

Article

Single-atom anchored g-C_3N_4 monolayer as efficient catalysts for nitrogen reduction reaction

Huadou Chai [a, b], Weiguang Chen [b], Zhen Feng [c], Yi Li [b], Mingyu Zhao [b] and Jinlei Shi [b], Yanan Tan[b,*], Xianqi Dai [a, *]

[a] School of Physics, Henan Normal University, Xinxiang, Henan 453007, China
[b] College of physics and Electronic Engineering, Zhengzhou Normal University, Zhengzhou, Henan 450044, China
[c] School of Materials Science and Engineering, Henan Institute of Technology, Xinxiang, Henan 453000, China
* Correspondence: author, E-mail address: xqdai@htu.edu.cn (X. Dai), yntang2010@163.com (Y. Tang).

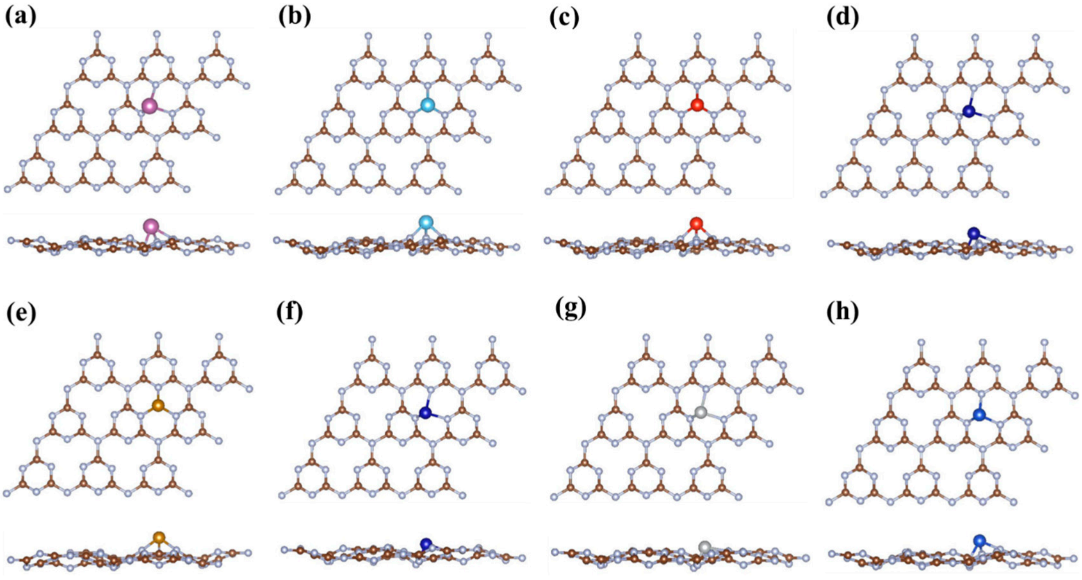

Figure S1. (a)-(h) Stable configurations of TM@g-C_3N_4 (TM= Sc, Ti, V, Cr, Fe, Co, Ni, Cu).

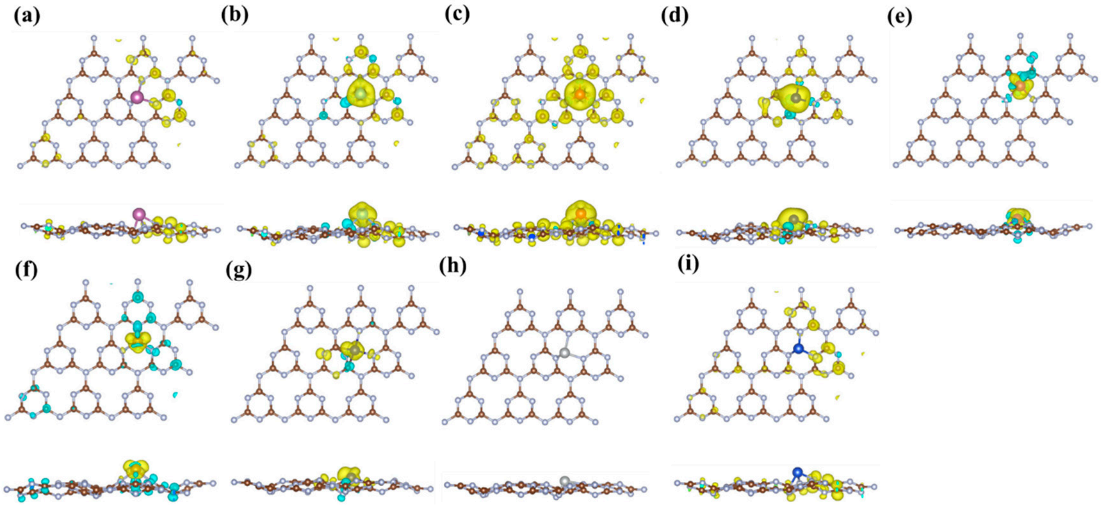

Figure S2. (a)-(i) Spin electron distributions of TM@g-C_3N_4 (TM= Sc-Cu) with isosurface 0.004 e/$Bohr^3$. Yellow and blue represent spin-up and spin-down states.

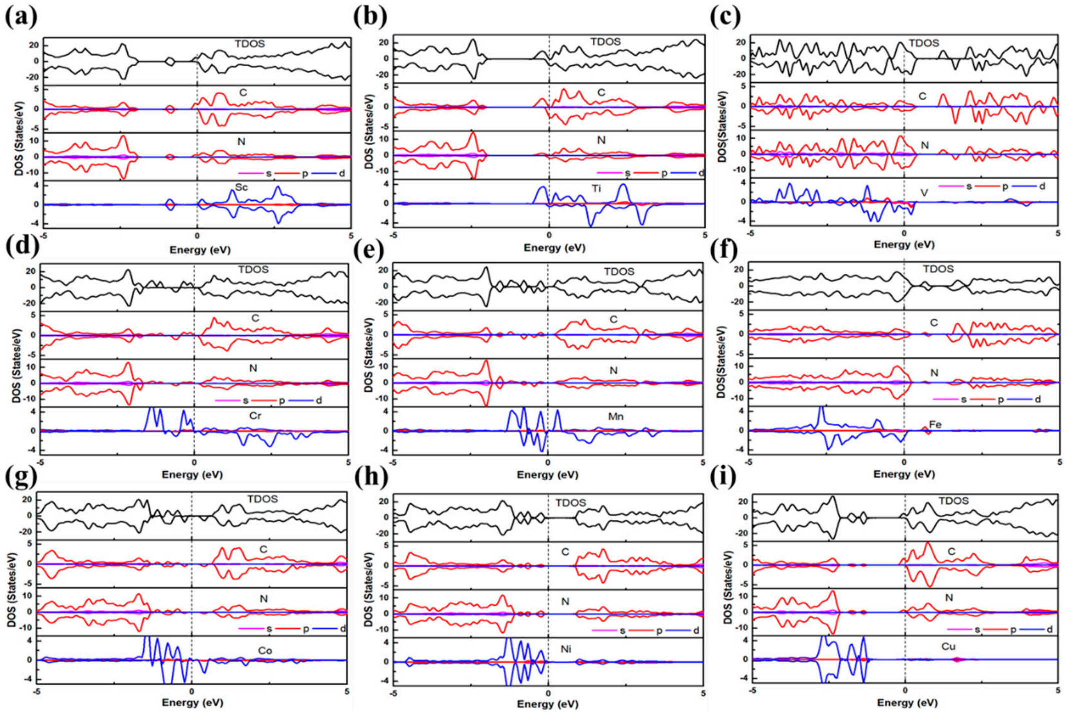

Figure S3. (a)-(i) DOS of TM@g-C_3N_4 (TM= Sc, Ti, V, Cr, Mn, Fe, Co, Ni, Cu). The Fermi level is set to zero.

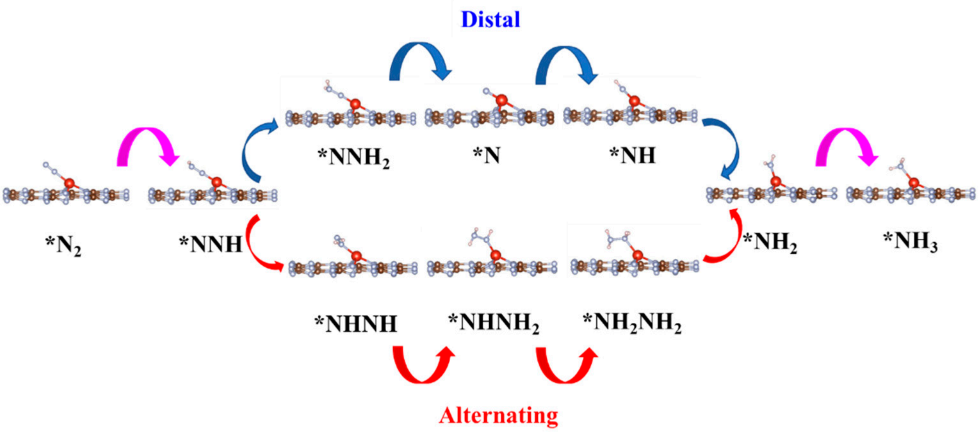

Figure S4. Optimized adsorption structures of intermediate via the alternating and distal mechanisms.

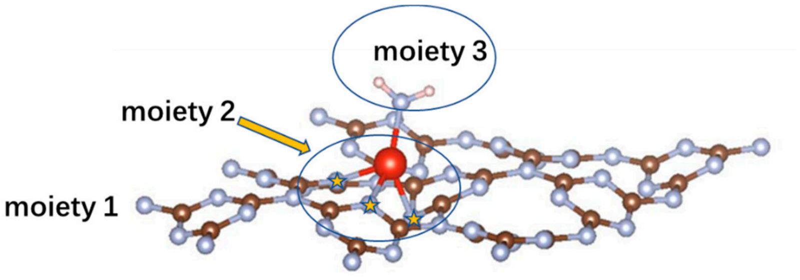

Figure S5. Diagram of the moiety 1(g-C_3N_4), moiety 2 (V-3N) and moiety 3 (the adsorbed intermediate N_xH_y).
